# Implementation planning for community-based point-of-care HIV testing for infants: Recommendations from community leaders in Kenya

**DOI:** 10.1371/journal.pone.0240476

**Published:** 2020-10-15

**Authors:** Lynton W. Macharia, Catherine Wexler, Melinda Brown, May Maloba, Ruby Angeline Pricilla, Natabhona M. Mabachi, Elizabeth Muchoki, Shadrack Babu, Martin Ochieng, Brad Gautney, Kathy Goggin, Sarah Finocchario-Kessler

**Affiliations:** 1 Department of Family Medicine, University of Kansas Medical Center, Kansas City, KS, United States of America; 2 Global Health Innovations, Nairobi, Kenya; 3 Christian Medical College, Vellore, Tamil Nadu, India; 4 Kenya Medical Research Institute, Nairobi, Kenya; 5 Global Health Innovations, Dallas, TX, United States of America; 6 Health Services and Outcomes Research, Children’s Mercy Hospitals and Clinics, Kansas City, MO, United States of America; 7 Schools of Medicine and Pharmacy, University of Missouri-Kansas City, Kansas City, MO, United States of America; Waseda University, JAPAN

## Abstract

**Background:**

Early infant diagnosis (EID) establishes the presence of HIV infection in HIV-exposed infants and children younger than 18 months of age. EID services are hospital-based, and thus fail to capture HIV-exposed infants who are not brought to the hospital for care. Point-of-care (POC) diagnostic systems deployed in the community could increase the proportion tested and linked to treatment, but little feasibility and acceptability data is available.

**Methods:**

Semi-structured interviews (n = 74) were conducted by a Kenyan team with community members (Community Health Workers/Volunteers [CHW/CHV], Traditional Birth Attendants [TBAs], community leaders) and parents of HIV-exposed infants at four study sites in Kenya to elicit feedback on the acceptability and feasibility of community-based POC HIV testing.

**Results:**

Participants described existing community health resources that could be leveraged to support integration of community-based POC HIV testing; however, the added demand placed on CHW/CHV could pose a challenge. Participants indicated that other potential barriers (concerns about confidentiality, disclosure, and HIV stigma) could be overcome with strong engagement from trusted community leaders and health providers, community sensitization, and strategic location and timing of testing. These steps were seen to improve acceptability and maximize the recognized benefits (rapid results, improved reach) of community-based testing.

**Conclusion:**

Community members felt that with strategic planning and engagement, community-based POC HIV testing could be a feasible and acceptable strategy to overcome the existing barriers of hospital-based EID.

## Introduction

Early infant diagnosis (EID) services in Kenya were established as part of the national prevention of mother-to-child transmission (PMTCT) program. The primary objective of EID is to promptly identify HIV-positive infants and initiate lifesaving ART as soon as possible [1, 2]. This is particularly important given the significant reduction in mortality when HIV positive infants are started on ART prior to 12 weeks of age [3]. EID is offered free of charge in more than 190 centers including private health centers, government clinics and public health care institutions [2].

In 2014, 61% of live births in Kenya were delivered in a health facility, of which 46% were in a hospital [4]. More than one-third of births (37%) were delivered at home [4]. HIV-exposed infants are at higher risk for acquiring HIV when delivery occurs outside of health facilities and early infant HIV prophylaxis is missed [5]. It is crucial to reach these infants with early testing and linkage to treatment if needed. However, limited resources, high levels of HIV stigma, low levels of knowledge regarding PMTCT/EID services [2, 6] contribute to only about two-thirds of HIV-exposed infants receiving critical EID services [7]. Among mothers and caregivers who bring their infants for facility-based EID, many experience long delays in the notification of their infants test results [8] or may never be notified nor successfully linked to needed treatment, care and support [9].

Home and community-based HIV testing are feasible in low resource settings and offer the potential to increase testing coverage, identify more people living with HIV, and successfully link them to care [10–13]. In the past, community and home-based HIV testing was limited to older children and adults, since more complex HIV DNA PCR testing is required for definitive HIV diagnosis for infants and young children. In 2016, the World Health Organization (WHO) approved GeneXpert and Alere q for infant HIV POC testing [14, 15]. Manufacturers are developing smaller, mobile POC testing platforms that can enable community-based infant HIV testing, with same day results [16, 17].

Facilitating engagement of more HIV-exposed infants outside of the clinic is consistent with the Kenyan Ministry of Health (MOH) plan for differentiated HIV care in community settings, but little is known about the barriers and facilitators of doing POC testing outside of hospitals [18]. This pilot study provides formative findings from key community members and parents living with HIV regarding the feasibility and acceptability for community-based POC testing for HIV-exposed infants in Kenya.

## Methods

We conducted 74 semi-structured interviews with key community stakeholders: community health workers/volunteers (CHW/CHV), traditional birth attendants (TBAs), religious leaders, HIV+ male champions, respected community leaders (religious leaders, clan elders), and parents living with HIV. The study team worked with the clinical staff, mentor mothers, and community liaisons at the four study sites in rural communities (Central province, Coast province, and two in Nyanza province) to identify and engage key community members for participation (10 per site, 40 total). HIV+ parents (23 mothers, 6 fathers, 3 mother-father pairs; 35 total) accessing EID care for their infants were also interviewed to elicit their opinions regarding community-based POC testing for infants. Parents who had an HIV-exposed infant under 18 months of age and presented for EID care at the study hospital were eligible for inclusion in the interviews. Parent participants were recruited by each hospital’s mentor mothers, who have an established relationship with clients. Community members were eligible for inclusion if they fell into one of the a priori roles identified in community-based HIV care delivery including community health workers, traditional birth attendants, community leaders, religious leaders, or HIV+ champions in a single rural community within the study health facility’s catchment area. Convenience sampling was used in which all parent participants were engaged in care at the study hospitals and all community participants were known to clinical staff. Interviews were conducted at one of the four study hospitals that were accessible to the communities. Written informed consent was obtained from all study participants. Table 1 shows the distribution of participants by participant type and location, along with the roles of various groups.

**Table 1:** Description and distribution of participant type included in this study.

	Role	Central	Coast	Nya1	Nya2	Total
Community health worker/ volunteer (CHW/CHV)	Lay health workers who provide outreach services and serve as a direct liaison between health facilities and households.	5	4	7	5	21
Traditional Birth Attendants (TBA)	Lay health workers who provide education, referral, home-based antenatal care and deliveries	2	1	3	3	9
Male Champion (MC)	HIV-positive male peers who engage with HIV-positive men to support testing and HIV care	2	0	0	1	3
Community Leader (CL)	Well-respected individuals and opinion leaders of the community	0	1	0	1	2
Religious Leaders (RL)	Imams or pastors	1	2	0	1	4
HIV+ parent	HIV+ parents engaged in PMTCT and/or EID care at study hospitals	11	2	12	10	35
Total		21	10	22	21	74

Interviews were designed to elicit feedback on the acceptability and feasibility of community-based POC HIV testing. Topics covered included perceived benefits, facilitators and barriers of community POC testing, recommendations for optimal implementation of community-based POC testing, and linkage to HIV services. Interviews were conducted in English, Swahili or participants’ preferred local language in a private setting and last approximately 30–45 minutes. All interviews were conducted either by the trained study coordinator (MO or SB) or by the in-country co-PI (MM), who had no prior contact with the study participants. Audio recordings were translated into English, and transcribed. Each participant was assigned a unique study identification number, and all identifying information was excluded from interview transcripts. Three analysts (LM, MB, RP) independently coded transcripts for a priori (perceived benefits and barriers of community-based POC, suggestions for implementation) and emergent themes and met periodically to develop a codebook through an iterative consensus building process using Dedoose. The initial codebook was subsequently refined for key themes relevant to the study. All participants provided written, informed consent prior to participation. Ethical human subjects research review and approval was obtained from the Kenya Medical Research Institute (SSC3390) and the University of Kansas Medical Center (STUDY00140399).

## Results

### Perceived benefits of community-based POC testing

Community members and parents noted several expected benefits of community-based POC testing. These benefits included broadening coverage, streamlining EID, and reducing parental burden. Participants felt that the primary benefit of community based POC testing would be increased coverage of HIV testing among infants and children, *“Some mothers will give birth*, *but may not see a need of taking their newly born infants to hospital*. *Some infants don’t have a hospital card at all…Such individuals need to be visited at home*, *so that all who turn positive*, *can be linked into care services” (MC1_Central)*. In particular, community-based POC was seen to benefit the “*many mothers who fear going to the hospital*” *(Parent5*, *Nyanza1)* due to stigma, or those who might wait until symptoms appear to come for testing, “W*hen we tell them to come for testing at the facility*, *they will argue that there is no need*, *it’s until [the child] gets sick” (CHW2_Coast)*.

POC testing in the community would also allow rapid diagnosis by streamlining the EID cascade and eliminating steps that contribute to long turnaround times: *“I feel the device* [Gene-X and Alere-Q] *will be useful since we have been referring from the community to facility*, *then to* [the hospital], *then they [blood samples] are taken to Nairobi*.*”* (CHW1_Coast)

More rapid turnaround time would also reduce the burden of EID participation, especially for those living far from health facilities: “*With the current PCR testing procedures*, *individuals spend a lot of time coming to and fro*, *to check on their infant’s results*. *But for this*, *you know the health of your child early”* (CHW1_Coast), and ameliorate mothers’ anxiety around the status of their infants “*because when one is tested*, *and they wait for the results one can even get depressed…because they do not know how the results will be*.*”* (RL1_*)*

### Perceived barriers to community-based POC testing

Despite these perceived benefits, participants anticipated several key challenges to introducing community-based POC testing, including HIV stigma, fear of disclosure, and high CHW workload.

Nearly all participants mentioned stigma and fear of disclosure as major barriers to HIV services, including community-based POC testing: *“There is so much stigma in the homesteads*. *People shun from disclosing about their HIV status*, *for fear of being discriminated*.*”* (MC2_Central). Concerns with confidentiality were pervasive throughout the testing process with parents first worried that they’d be seen accessing the test for their infant, which would indicate their own HIV+ status, and then worried that their infant’s result would be shared with others: *“if mistakenly one person knows that your home has been visited by those doing the test that person will tell another person and that way information will spread”* (Parent5_Central)

Furthermore, community members described how stretched health resources could hinder community-based outreach and linkage to care. CHW, the primary link between communities and the facility, are limited by their catchment area geographic distribution and high client loads “*A CHW gets assigned about 200–300 people*, *of which is not possible for them to reach them all… Some clients live in far places*, *which can be difficult to reach*.” (TBA1_Nyanza2).

### Logistics for implementing community-based POC testing

Community members and parents identified existing resources and recommended strategies for implementing POC.

### Leveraging existing resources

Participants emphasized the importance of involving community members in the planning and implementation of community-based POC testing. Due to a lack of trust in outside medical interventions or government health initiatives, community leaders’ endorsement of community-based POC testing was seen as essential to increase acceptance and ownership:

“*[Sensitization] should involve the key people in the village e*.*g*. *the community health volunteers*, *the village elders…we now have ward administrators*, *and the religious leaders*. *If you bring them on board and inform them…they will take the information back to their people and there will be no speculation*.*”* (CHW1_Coast).

“*[Include] the CHEWS*, *the community health extension workers*. *The CHC*: *the community health committee…administration*, *police or the village elders*. *And then*, *the community health workers so that they own the project from the community” (CHW2_Nyanza1)*.

Participants suggested training all levels of health care workers (paid and volunteer) about POC HIV testing to support mobilization, testing, and follow up care.

“*With good mobilization strategy I will be able to know where each and every one comes from*, *it would be the responsibility of the CHW to do follow ups on clients so that they adhere… Inform each of the service providers at the facility about POC*, *then also remember to include CHWs and TBAs for successful mobilization and implementation*”(TBA3_Nyanza1). Consistent with their existing activities to promote HIV testing to the communities and provide HIV-related training to health professionals, participants described how non-governmental organizations (NGOs) could be leveraged for community-based POC training and sensitization. However, the extent of NGO activities in more rural areas was uncertain, *“In the towns I know there are NGOs but in the villages am not sure*.*”* (RL1_Central)

Participants noted the training for personnel conducting community based POC testing should cover counseling, retesting, referral, and maintaining confidentiality, “*the people conducting the tests should be told in advance that they must maintain confidentiality at all times*” (CHC4_Central). In addition, they suggested additional training for all involved with sensitization to ensure consistent and correct messaging, “*I feel we need…refresher courses to the church leaders*, *health workers and also the administration if possible…because unless they get the right information they can be giving their own information which will be wrong (RL1_Central)*

### Priority population for testing

While participants felt that infants and children not currently enrolled in health systems were a priority population for POC testing, they emphasized the need to make testing available to a broader population. Furthermore, broadening coverage to include the general population was seen as a strategy to minimize stigma and the potential for unintentional disclosure: “*You should not selectively pick the household with infants*, *and ignore those with no infants*. *Doing so will leave people wondering; ‘Why did they only go to that household*?*’…Community screening should be generalized*, *even when you are targeting a specific population*” (MC1_Central). This could be done through offering a range of integrated services or by incorporating POC testing into existing community health initiatives–specifically vaccination campaigns- to support uptake: *“It’s just by incorporating it during the polio vaccination campaigns*, *where most mothers normally bring their children to get vaccinated*. *This can be the best time to counsel them into accepting to take the test*.*”* (Parent2_Coast)

### Location of testing

There was mixed feedback about the preferred location of testing, with varied recommendations to use temporary tents set up in public places (schools, churches, chief’s area), home-based POC testing, or hospitals, Table 2.

**Table 2:** Perceived benefits and barriers of various testing locations.

Suggested location for POC testing	Primary benefits of this location	Possible challenges with this location
Public places	• High visibility and awareness	• Low turnout • Fear of stigma
Home-based	• Increased confidentiality compared with public places • Increased patient comfort • Established relationship with CHW/CHV conducting outreach • Proven success in other initiatives	• Involuntary disclosure to family or neighbors • Stigma if certain homes singled out • Mistrust if testing offered by community outsiders
Hospital	• High level of confidentiality • Acceptable visits (well child clinic) thus less stigma • Provision of high-quality services	• Travel time and cost • Perception that hospitals are only for when you’re sick

### Public places

Some community leaders suggested utilizing public places for health education and services, *“I think in the villages we need to have some locations that can be set like in the chief’s area*, *in the health center*, *wherever there is a dispensary and even in the shopping center*.*”* (CHW2_Nyanza1). They suggested “t*o bring snacks and refreshments*, *organize for tents*, *chairs*, *educate and provide transport*” (TBA3_Nyanza2) to provide incentives and minimize barriers.

If conducted in a public setting, tents were seen as acceptably private: “*you can put up your tents*, *and make sure that they are covered properly*. *Those inside*, *should be 2 or 3 people only*, *the mother of the child and the doctor that is attending to her*, *only*. *I think that would be safe*.” (Parent4_Nyanza1)

However, some expressed concern about POC HIV testing in public spaces. One TBA felt that public places might result in low turnout, *“People cannot come at schools*. *It is only when you are walking door to door*, *that their response will be high*. *If you are going to wait for them to come at schools*, *nobody will show up*.*”* (TBA1_Nyanza2)

### Home-based testing

Many preferred a home-based POC testing strategy to capture infants and their families who do not utilize health facilities because, “*If you take them door to door*. *They welcome the services because with HIV because they think if you bring it to the house*, *it is really confidential to them*.*” (CHW4_Nyanza1)*

However, others thought that door to door testing would not be well accepted, *“Some people will not take it well*, *they will be like; “How did you know I was in need of those services*? *What intentions do you have*? *Please leave my house*!” (Parent2_Coast).

Parents also expressed concerns that door to door testing may risk exposure of mother’s HIV status if she had not yet disclosed to families and emphasized the need for strict confidentiality, *“If you will do the house to house testing before doing the test first inquire from her if she has disclosed to the spouse so that undue disclosure does not happen*. *If she has not disclosed then you will have to postpone the testing and find other ways to ensure the test is done*.*”* (Parent1_Central)

### Hospitals

Others expressed support for stationing the POC testing in hospitals. These participants felt that hospital-based testing would best assure confidentiality.

“*The hospital is a bit more confidential than doing it in the community*…*You will find some mamas are not ready to be tested in the community*. *Testing*, *they don't like it mostly in the community*.*” (Parent9_Nyanza2)*

Furthermore, they felt that standard hospital-based testing would facilitate better counseling and more rapid treatment, in case of a positive result:

“*Such a service should be offered in a hospital set up*, *where supportive counselling can be given*, *and individuals can walk home feeling relieved and well aware about their appointment*.*”* (MC1_Central)

*I do not see the need to go to the community it is better to do things as they are being done now*. *Testing to be done at the hospital and they are started on treatment* (Parent4_central)

### Linkage to care

For all who received an HIV positive result via POC testing in the community, participants described an existing system for referrals to the facility and for CHW/CHV follow-up, *“[Linkage is] so easy because we already have CHVs on the ground*, *whom their roles are linkages and referrals*. *The CHV can be alerted and given the necessary information*, *so that he can conduct a home visit*.” (MC1_Central). Counseling was seen as a key component of successful linkage to care, “*It’s just … adherence counselling*, *once they turn out positive*. *We should encourage them by telling them; “This is not a death sentence*, *if you adhere well*, *you will live long…once the virus is suppressed*, *you can live your normal life” (CHW1_Coast)*.

## Discussion

Key themes emerging from these formative interviews with key stakeholders highlighted considerations for the implementation of community-based POC testing for infants. These included leveraging existing community resources to maximize uptake, utilizing community leaders and known community health workers for strategic planning and implementation to increase ownership and mitigate mistrust of externally initiated health efforts, and ensuring confidentiality through strategic location selection and adequate staff training. Similar strategies have increased successful implementation of home-based HIV testing among older children and adults [10].

Reaching pregnant and postpartum women who do not seek routine services at health care facilities or who give birth at home is critical to optimize early infant diagnosis, treatment, and HIV prevention. POC testing at the community level can further expand EID coverage by reaching infants born at home who do not utilize hospital-based care and fail to be linked to much needed HIV testing and treatment in a prompt manner. In order to reach this target population, the consensus has been to test the general population (infants, parents and other family members). Participants identified two primary reasons why testing the entire community was preferable to targeted infant testing. First, they felt that it would reduce the potential for unintentional disclosure. Indeed, previous literature suggests a “whole community” approach to HIV testing can reduce stigma associated with testing [19]. Second, participants noted that a whole community approach would increase the overall proportion of community members who know their HIV status. Parental consent for their child to be tested for HIV in the community was found to be lower than consent for their own HIV testing [10, 11, 20], thus, by offering testing to adults and children, implementers are likely to identify a larger number of at-risk infants and children. Since older children and adults can be tested using less expensive antibody tests, offering antibody testing to all adults may be a cost-effective strategy of identifying at-risk infants (i.e. those whose mothers are either HIV-positive or have an unknown HIV status) who would most benefit from community-based POC testing [10].

Stigma remains a pervasive fear that needs to be carefully considered in any strategy to introduce infant POC testing within the community. These concerns influenced recommendations on where to test, personnel to be involved, and priority populations to be tested. Competing preferences for privacy and community-wide coverage led to competing recommendations for both home-based and public location testing within the community. Previous research indicates that home-based testing, compared to testing at community locations, results in similar uptake among adolescents and adults and higher uptake among younger children (<12 years) and first-time testers [20], indicating that home-based testing may be the preferred strategy to reach very young children.

The varying recommendations from stakeholders in our study indicate that efforts to increase HIV testing need to address differing needs and preferences of various populations. This can be achieved by incorporating complementary testing strategies, such as offering community-based testing at both public places (such as schools, churches, etc) and homes, while continuing to encourage hospital engagement could maximize the reach of community-based testing initiatives. For those diagnosed with HIV in the community, stigma remains a challenge for linkage to facility-based ART, though community networks to provide pre and post-diagnosis counseling were seen as a key strategy to address these concerns. Offering multiple options for treatment initiation including hospital referrals, facilitated linkage to ART care [21], and community-based ART programs [22] may further improve treatment uptake and retention. However, further research and dialogue with community members will be needed to ensure context appropriate implementation of testing and linkage in each community.

Participants in this study emphasized the need for training for all involved with introduction of POC HIV testing. Despite labeling CHW/CHV as vital for outreach and linkage to care efforts, participants also acknowledged existing high workload burdens and indicated possible concerns with CHW/CHV maintaining patient confidentiality. Other studies in Kenya have noted that, while community health workers can improve the reach and uptake of HIV services, including home-based testing [23, 24], community members hold concerns regarding CHW/CHV’s ability to maintain confidentiality and the quality of information and services that they provide [25, 26]. This further highlights the need to train to all personnel involved in community-based HIV testing in HIV diagnosis and treatment protocols; including strategies to maintain confidentiality and privacy during home visits and communication with families.

Of vital importance of any home-based testing strategy is maximizing linkage to care among those who test HIV+. Community members were confident that the existing system of CHW referral would simplify infant linkage to care after a positive diagnosis. However, a systematic review found that linkage to care after community-based HIV testing was low (33%) if a positive diagnosis was followed by referral only [27]. Given the unique challenges of infant and child HIV care [28], linkage to care may pose additional challenges for infants. Employing additional strategies, other than referral, to assist linkage to care (e.g, provision of transport funds; follow-up counselling, facilitation of the initial HIV clinic visits) have improved linkage to care after community-based HIV tests in adults; however their effect on ART initiation and care is unclear, and their impact on infant linkage has not been established [27].

### Strengths and limitations

The strengths of this study were having a Kenyan team well known to the community which allowed for easy recruitment of participants and representation of various types of community leaders. Knowledge of local languages by data collectors allowed interviews to be conducted in languages preferred by participants. Utilizing four different study regions in Kenya make our findings more generalizable. Limitations of this study include a relatively small sample size by region and by type of community leader and the inclusion of only parents already engaged in their infant’s EID care. Another limitation is that POC testing is a new technology that some may have had little exposure to, and this may have limited feedback.

## Conclusion

Our findings indicate a high level of acceptability for community-based POC among community members. In collaboration with local community leaders, health professionals, NGOs, community-based POC testing may be a feasible solution to address gaps in hospital-based infant HIV DNA PCR testing. To maximize success, implementers should offer a variety of testing locations (home-based vs testing sites set up at public spaces), provide comprehensive training to all personnel involved in testing, offer testing to the entire community, and develop systems to ensure confidentiality and assist with linkage to care for children and adults identified as HIV-positive. This project provides valuable information to researchers, stakeholders and respected community leaders regarding community-based POC introduction.

## Supporting information

S1 File (DOCX)
