# Supplementary material for: Implementation planning for community-based point-of-care HIV testing for infants: Recommendations from community leaders in Kenya
Source: PLoS One. 2020 Oct 15;15(10):e0240476. doi: 10.1371/journal.pone.0240476 (PMC7561120; doi:10.1371/journal.pone.0240476)
Supplement: S1 File — (DOCX) [file pone.0240476.s001.docx]

**SUPPORTING INFORMATION. KEY INFORMANT INTERVIEWS GUIDE: COMMUNITY MEMBERS**

**Introductory Questions***Before we talk about your thoughts on implementing these POC systems, I would like to ask you a little bit about your community.*

1. Could you tell me a bit about your role here in the community?
2. What are some things that motivate people to seek health care in your community?
   *Probe: What types of health concerns? Support from community members/leaders? Encouragement from family?*
3. From your perspective, what are some of the challenges that community members face in accessing health services?

*Probe: How far is the nearest health care facility? Nearest hospital? How is transportation into/out of the village? Hospital fees/bus fares?*

- 1. Are there any particular challenges associated with accessing HIV services, including testing and treatment services, PMTCT, and EID?
     *Probe: Would community members fear what others will think of them? Are community members willing to be tested/receive care?*
  2. How important do you think increasing access to HIV testing is in your community? Why?

**Questions about Existing Resources**

1. What resources exist in this community to help provide community members with health care services?
   1. Are there any dispensaries in the area? If yes, what services do they provide?
   2. Are there any NGOs that work in the area? If yes, what services to they provide?
   3. Are there community health workers in the area? If yes what services do they provide?
2. Are there any midwives or birth attendants that provide community-based maternity services to pregnant women and new mothers? If yes, could you describe the services that they provide?
3. What resources exist in the community that provide services to infants and children (i.e vaccinations, under-5 clinics, weight tracking)?
4. What resources exist in the community to provide HIV testing and treatment services to community members?
   *Probe: Where can people go to get tested for HIV? How far away are the nearest services?*
5. How aware are community members of these resources? How commonly are they used by the community? What is the quality of care provided?

*Probe: For MCH resources (specific to infant testing)? For HIV resources?*

**Questions about Using POC tests for community-based testing**

*Now, I would like to ask you about your thoughts on using POC testing systems for periodic community-based HIV testing.*

1. In your opinion, what would be some benefits of using POC systems to bring HIV testing to the community?
2. From your perspective, what would be some challenges associated with providing periodic, community-based HIV screening using portable testing systems?

Probe: Issues of confidentiality? Issues of stigma?

1. What are some ways that each of these challenges can be minimized?
2. If point of care HIV testing was available in your community, how would you expect community members to respond?

*Probe: Would community members be comfortable accessing these services? Why or why not? What level of demand would you anticipate?*

1. Who would need to be involved in the planning and execution of community based POC HIV testing? And how do you foresee each of these people being involved?

*Probes: CHW roles? Dispensary/health clinic workers roles? Midwife/birth attendant roles?*

1. How do you foresee community based HIV testing using POC systems being implemented in the community?
   1. What would be the ideal location for this testing to occur? Why this location?
   2. With what frequency should POC HIV testing be made available to the community?
   3. Should community-based HIV testing using POC systems be offered for EID only or for everyone?
      *Probe: Are there specific concerns related to disclosure/stigma if POC is offered for EID only? For everyone?*
2. What would be the best ways to get the word out about the availability of POC HIV testing?
   1. What messaging would be necessary to increase community interest in HIV testing?
3. How can we ensure those who test positive receive appropriate follow up care?

*Probe: Post testing counselling? Availability of treatment services?*

- 1. What might be some of the challenges in providing support in these areas?
  2. What types of messaging could be helpful to encourage community members to receive care after receiving their test result? What is the best way to reach the community with these messages?

**Concluding Questions**

1. If mobile POC testing was available today, what would need to happen in your community to be prepared to start using it?
2. What other advice or suggestions do you have for successful introducing community based POC HIV testing?

**SUPPORTING INFORMATION. KEY INFORMANT INTERVIEWS GUIDE: COMMUNITY MEMBERS**

**MWONGOZO WA MAHOJIANO YA KUTOA HABARI MUHIMU – WANACHAMA WA JAMII**

**JINA LA UTAFITI:**

**Maswali tangulizi**

Kabla tuongee kuhusu mawazo yako ju ya utumizi wa mbinu hizi za kupima virusi vya ukimwi, ninelipenda kukuuliza maswali kidogo kuhusu jamii yako.

1. Unaweza kunieleza kwa kifupi majukumu yako hapa kwa hii jamii?
2. Ni mambo kama yapi yanayoweza kuwahamasisha watu ili kutafuta huduma za afya katika jamii yako?

*Chunguza kwa kina: Ni aina gani zinaleta wasiwasi wa kiafya? Kuna usaidizi gani kutoka kwa wanachama wa jamii/viongozi? Kuna faraja gani kutoka kwa jamii?*

1. Kwa mtazamo wako, kuna changamoto gani ambayo wanachama wa jamii wanapitia wakitafuta huduma za afya?

*Chunguza kwa kina: Kituo cha afya kilichoko karibu kiko umbali kiasi gani? Hospitali iliyoko karibu Zaidi? Hali ya usafiri kuingia na kutoka kijijini iko vipi? Ada ya hospitali/nauli ya basi?*

- 1. Je, kuna changamoto hasa zinahosiana na kupata huduma za virusi vya ukimwi, ikiwemo huduma za upimaji na matibabu, huduma ya kuzuia uambukiza wa mtoto kutoka kwa mama,na huduma ya utambuaji wa virusi vya ukimwi kwa watoto wachanga?
     *Chunguza kwa kina: Je wanachama wa jamii wanahofia vile wengine watafikiria kuwahusu? Je, wanachama wa jamii wako tayari kupimwa virusi vya ukimwi/kupokea huduma?*
  2. Unafikiria ni muhimu kiwango gani kuongeza uwezekano wa kufikia huduma ya upmaji wa virusi vya ukimwi katika jamii yako? Kwanini?

**Maswali Kuhusu Raslimali Zilizoko**

1. Ni raslimali gani zilizoko katika jamii hii zinazoweza kuisaidia jamii kupatia wanachama wake huduma za kiafya?
   1. Je, kuna zahanati yeyote eneo hili? Kama ndio, ni huduma zipi wanazopeana?
   2. Je, kuna mashirika yeyote ya kibinafsi yanayofanya kazi eneo hili? Kama ndio, ni huduma gani zinazotolewa na mashirika haya?
   3. Je, kuna wahudumu wa afya wa jamii katika eneo hili? Kama ndio, ni huduma gani wanazopeana?
   4. Je, kuna wakunga wanaopatiana huduma za msingi wa kijamii kama vile huduma za uzazi kwa wanawake wenye mimba na waliojifungua majuzi? Kama ndio, unaeza kuelezea huduma zilizoko?
2. Ni raslimali gani zilizoko katika jamii ambazo zinaweza kusaidia kupeana huduma kwa watoto wachanga (kama vile chanjo, kliniki ya watoto chini ya miaka 5, kufuatilia uzito)?
3. Kuna raslimali gani katika jamii za kusaidia kupatia huduma za kupima virusi vya ukimwi na matibabu ya virusi hivyo kwa wanachama wa jamii?
4. *Chunguza kwa kina: Watu wanaweza kuenda wapi ili kupimwa virusi vya ukimwi? Kituo cha karibu Zaidi kiko umbali kiasi gani?*
5. Je, wanachama wa jamii wako na ufahamu kiasi gani kuhusu raslimali hizi? Raslimali hizi zinatumiwa vipi na jamii kwa kawaida? Huduma zinazotolewa zina ubora kiasi gani?

*Chunguza kwa kina: raslimali ya kliniki ya wajawazito (inayohusiana na upimaji wa watoto wachanga)? raslimali ya huduma za virusi vya ukimwi?*

**Maswali kuhusu matumizi ya mbinu za upimaji wa virusi vya ukimwi katika vituo vya huduma kwa upimaji wa msingi wa kijamii**

*Sasa, Naomba nikuulize mawazo yako kuhusu matumizi ya mbinu za kupima virusi vya ukimwi kwenye vituo vya huduma kwa madhumuni ya upimaji wa virusi hivi wa mara kwa mara katika jamii*

Kwa maoni yako, kuna manufaa gani haswa ya kutumia mifumo ya upimaji wa virusi vya ukimwi kwenye vituo vya huduma ili kuleta upimaji wa virusi hivi katika jamii?

1. Kulingana na mtazamo wako, ni changamoto kama gani zinazohusika na utoaji huduma wa mara kwa mara, na upimaji virusi vya ukimwi kwa msingi wa kijamii kutumia mifumo iliyo rahisi kusafirisha au kuhamisha?

Chunguza kwa kina: Maswala ya usiri? Maswala ya unyanyapaa?

1. Njia gani zinazoweza kutumiwa kupunguza changamoto hizi?
2. Iwapo mbinu hizi za kupima virusi vya ukimwi kwenye vituo vya huduma zingelikuwepo katika jamiiyako, ungelitarajia wanachama wa jamii yako kushiriki?

*Chunguza Kwa Kina: Je, wanachama wa jamii wangeliona vizuri kupata huduma hizi? Kwanini wangefurahia/kwanini hawangefurahia? Ni kiwango gani cha uhitaji ungetarajia?*

1. Ni nani angehitajika kuhusishwa na mipangilio na utekelezaji wa upimaji wa virusi vya ukimwi katika msingi wa kijamii? Kwa mtazamo wa mbeleni unaona watu wakihusika kwa njia gani?

*Chunguza kwa kina: Majukumu ya wahudumu wa afya wa kijamii? Zahanati/Majukumu ya wahudumu wa afya? Majukumu ya wakunga?*

1. Kwa mtazamo wa mbeleni, unaona mbinu hizi za kupima virusi vya ukimwi kwenye vituo vya huduma zikitumika katika jamii?
   1. Ni sehemu gani bora upimaji huu wa virusi vya ukimwi unaweza kufanyika? Kwanini sehemu hiyo?
   2. Huduma hii ya kupima virusi vya ukimwi kutumia mbinu kwenye vituo vya huduma inafaa kufanyika mara ngapi katika jamii?
   3. Je, huduma ya upimaji wa virusi vya ukimwi wa ki msingi wa jamii kutumia mbinu za kupima virusi vya ukimwi kwenye vituo vya huduma inafaa kupatiwa watoto wachanga pekee ama kila mtu?
      *chunguza kwa kina: Je, kuna wasiwasi maalum kuhusiana na kutoa taarifa/unyanyapaa iwapo* mbinu za kupima virusi vya ukimwi kwenye vituo vya huduma *zikitolewa kwa watoto wachanga pekee? Kwa kila mtu?*
2. Ni njia zipi mwafaka zinazoweza kutumiwa kusambaza habari kuhusu kuwepo kwa mbinu hizi za kupima virusi vya ukimwi kwenye vituo vya huduma?
   1. Ni ujumbe gani muhimu unaweza kuongeza hamu ya jamii wajitokeze kwa wingi ili kupimwa virusi vya ukimwi?
3. Tunawezaje kuakikisha kwamba wale wanaopatikana na virusi vya ukimwi wanapata huduma ya ufuatilizi unaostahili?

*Chunguza kwa kina: Ushauri nasaha maada ya kupimwa? Kuwepo kwa huduma za matibabu?*

- 1. Ni changamoto gani zinazoweza kuonekana katika kutoa usaidizi katika maeneo haya?
  2. Ni ujumbe gani unaweza kusaidia kuwatia moyo wanachama wa jamii kupokea huduma baada ya kupokea matokeo ya kupimwa virusi vya ukimwi? Ni njia gani bora zinazoweza kutumiwa kufikisha jumbe hizi kwa jamii?

**Maswali ya kuhitimisha**

1. Iwapo mbinu za kupima virusi vya ukimwi katika vituo vya huduma zinazoweza kuhamishwahamishwa zingelikuwepo leo, ni nini ingehitajika kufanyika katika jamii yenu ili kuwa tayari kuanza kuzitumia?
2. Ni ushauri gani au maoni gani uliyo nayo yanayoweza kufanikisha kuanzisha mbinu za kupima virusi vya ukimwi katika vituo vya huduma kwa msingi wa kijamii?
